# Rice bran triterpenoids improve postprandial hyperglycemia in healthy male adults: a randomized, double-blind, placebo-controlled study

**DOI:** 10.29219/fnr.v62.1412

**Published:** 2018-10-02

**Authors:** Koichi Misawa, Hiroko Jokura, Akira Shimotoyodome

**Affiliations:** 1 Biological Science Laboratories, Kao Corporation, Ichikai-machi, Haga-gun, Tochigi, Japan; 2 Lifestyle Research Center, Kao Corporation, Tokyo, Japan; 3 Health Care Food Research Laboratories, Kao Corporation, Tokyo, Japan

**Keywords:** blood glucose, GIP, GLP-1, insulin, metabolism

## Abstract

**Background:**

Compared to white rice, brown rice induces a lower glycemic response in healthy and diabetic humans. This effect is partly attributed to the higher amounts of water- or oil-soluble bran components and dietary fiber in brown rice. We hypothesized that dietary supplementation with oil-soluble rice bran triterpenoids (RBTs; triterpene alcohol and sterol prepared from rice bran) might reduce the incidence of postprandial hyperglycemia in healthy humans.

**Objective:**

We examined the acute effects of a single RBT-supplemented meal on the postprandial blood glucose responses of healthy male adults in a double-blind, randomized, placebo-controlled, crossover trial.

**Design:**

Nineteen subjects consumed a test meal containing either placebo- or RBT-supplemented olive oil. Blood biomarkers were evaluated in a fasting state and up to 240 min postprandially.

**Results:**

Compared to the placebo-supplemented meal, the RBT-supplemented meal significantly suppressed the increase in postprandial blood glucose level. A subclass analysis revealed that RBT-supplemented oil significantly reduced blood glucose increases in subjects with higher postprandial blood glucose elevations. Postprandial increases in blood insulin, glucose-dependent insulinotropic peptide (GIP), and glucagon-like peptide-1 (GLP-1) levels did not differ between the groups.

**Conclusion:**

These results suggest that RBT consumption improves postprandial hyperglycemia in healthy humans, especially those with higher postprandial glucose increases.

Diabetes, a condition that results from impaired insulin secretion or insulin resistance, is currently a health problem worldwide. Global statistics suggest that 425 million people were living with diabetes in 2017 and predict that this number will exceed 629 million by 2045 (1). Hyperglycemia is a major risk factor for both microvascular and macrovascular complications (2, 3). Recent epidemiological studies indicated an association between isolated postprandial hyperglycemia and increased cardiovascular mortality (4–6). Therefore, lifestyle changes that ameliorate/prevent postprandial hyperglycemia, and particularly dietary changes, are recommended as primary preventive and treatment measures. Accordingly, the demand for food components that can prevent/mitigate postprandial hyperglycemia in diabetic, pre-diabetic, and healthy humans has increased.

For thousands of years, rice has been consumed as a staple food in Asian countries. Unpolished brown rice contains various nutrients in the germ and bran layers, which are removed during the production of white rice. Recent increases in the incidence rates of diabetes and other relevant diseases have led to increased interest in brown rice and its components as natural foods with significant health benefits. Rice bran contains nutrients such as minerals, fiber, fatty acids, and oil-soluble nutraceuticals (7). Triterpene alcohols, plant sterols, and their ferulic acid esters (γ-oryzanol), which exhibit hypocholesterolemic (8), antioxidant (9), anti-inflammatory (10), anti-carcinogenic (11), and other effects, are characteristic components of rice bran oil.

We previously demonstrated that rice bran triterpenoids (RBTs; triterpene alcohol and sterol prepared from rice bran) lowered postprandial blood glucose (12) and increased glucose-dependent insulinotropic peptide (GIP) (13) levels in mice. GIP is a gut hormone secreted from enteroendocrine K cells into the bloodstream after food consumption (14). Dietary carbohydrates and fats are major secretagogues for postprandial blood GIP responses (14). Our previous study suggested that cycloartenol, a major triterpene alcohol derived from RBTs, reduced postprandial intestinal glucose absorption by suppressing the translocation of sodium-glucose cotransporter-1 (SGLT1) to the apical plasma membranes of intestinal epithelial cells (12).

Compared to milled rice, brown rice induces lower glycemic responses in healthy and diabetic humans. This effect is partly attributed to the relatively higher amounts of water-soluble rice bran components (phytic acid and polyphenols), dietary fiber, and oil in brown rice (15). However, the acute effects of rice bran-derived oil-soluble components on postprandial blood glucose response remain to be clarified.

Against this background, we hypothesized that dietary supplementation with RBTs might reduce postprandial hyperglycemia in healthy humans. To test this hypothesis, we examined the acute effects of a single RBT-supplemented meal on the postprandial blood glucose responses of healthy male adults in a double-blind, randomized, placebo-controlled, crossover trial.

## Materials and methods

### Materials

Commercially available food-grade RBTs (Oryza triterpenoid – PK) were purchased from the Oryza Oil & Fat Chemical Company (Aichi, Japan). The triterpenoid composition was determined using gas chromatography (Agilent 6890N series GC System; Agilent Technologies Inc., Santa Clara, CA, USA). This composition included cycloartenol, 24-methylene cycloartenol, β-sitosterol, and campesterol in mass ratios of 57/38/1/2% (w/w) (Lot Q-404). Rice (Sato No Gohan^®^; energy 294 kcal, carbohydrate 67.8 g, protein 4.2 g, fat 0 g) was obtained from Sato Foods (Niigata, Japan).

### Subjects

**Fig. 1:**
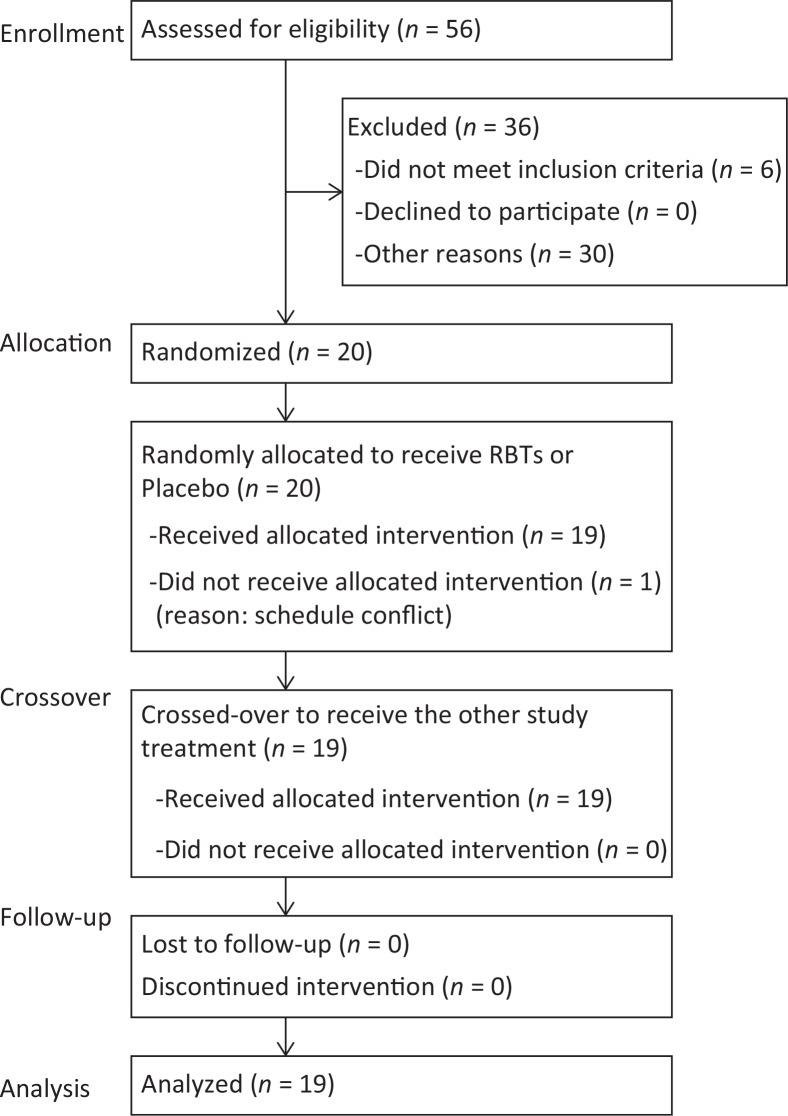
Flow chart of the study and numbers of study participants from screening to study completion. RBTs, rice bran triterpenoids.

Twenty of the 56 men initially recruited for eligibility screening were enrolled and 19 subjects aged 37–56 years were analyzed in this study (Fig. 1). The subjects were required to meet the following inclusion criteria: male sex, body mass index (BMI) between 20 and 28 kg/m^2^, systolic blood pressure ≤139 mmHg and diastolic blood pressure ≤89 mmHg, and blood triglyceride (TG) concentration between 50 and 149 mg/dL. The main exclusion criteria were as follows: chronic pharmaceutical intake; diagnosed heart, liver, kidney, gastrointestinal, or infectious disease; diagnosed food allergy; smoking habit; alcohol abuse; and simultaneous participation in another study. The study interventions were conducted at the Osaki Hospital Tokyo Heart Center (Tokyo, Japan).

We calculated the sample size based on data from a preliminary pilot study. The previous data indicated that the differences in the responses of matched pairs were normally distributed, with a standard deviation of 15. Accordingly, if the true difference in the mean responses of matched pairs was 10, we would need to study 20 pairs of subjects to be able to reject the null hypothesis of a response difference of zero, with a probability (power) of 0.8. A 0.05 probability of a type I error was associated with the test of this null hypothesis.

The Human Ethics Committee of Kao Corporation approved the study protocol. All subjects provided written informed consent. The study was conducted under the supervision of the chief investigator in accordance with the tenets of the Declaration of Helsinki. The trial was registered at UMIN Clinical Trials Registry (UMIN000017447; preregistered on May 7, 2015).

### Methods

#### Study design and diet

We examined the acute effects of the ingestion of a single meal containing RBT-supplemented olive oil on blood glucose levels in a double-blind, randomized, placebo-controlled, crossover trial with a washout period of 7 days. After an overnight fasting period of at least 10 h, fasting blood samples (baseline) were collected. Subsequently, the subjects were requested to consume the assigned meals. Blood samples were also collected at 0.5, 1, 2, 3, and 4 h after the beginning of the meal consumption. The subjects were not allowed to consume any food or beverages for 4 h after meal intake, except for 200 mL of mineral water at the 2-h time point. The subjects consumed one of the following two meals: 1) placebo (200 g of cooked rice containing 11 g of olive oil) or 2) RBTs (200 g of cooked rice containing 11 g of olive oil supplemented with 16.5 mg of RBTs).

#### Blood sampling and analysis

Blood samples were obtained from the antecubital vein. Blood glucose levels were measured using a self-monitoring device (ACCU-CHEK COMFORT, Roche Diagnostics, Basel, Switzerland) immediately. At each time point, approximately 5 mL of blood was collected into Venoject^®^ II vacuum tubes (Terumo, Tokyo, Japan) and centrifuged at 3,000 g for 15 min at ambient temperature. Serum samples were collected and stored at −80°C until analysis. In addition, 2mL of blood was collected into BD Vacutainer™ plastic blood collection tubes containing EDTA 2K (Becton, Dickinson and Company, Franklin Lakes, NJ, USA) for plasma preparation. After adding a protease inhibitor (Protease Inhibitor Cocktail powder, P2714-1BTL, Sigma, St. Louis, MO, USA) and serine protease inhibitor (Pefabloc, Roche Diagnostics, Basel, Switzerland), the blood samples were centrifuged at 2,900 g for 10 min at ambient temperature. The resulting plasma samples were stored at −80°C until analysis. The blood concentrations of TG, total cholesterol, high-density and low-density lipoprotein cholesterol, aspartate transaminase (AST), alanine transaminase (ALT), and lactate dehydrogenase (LD) were measured using an automatic blood analyzer (ACCUTE TBA-40FR, Toshiba Co., Tokyo, Japan) and compatible reagents (Nittobo, Tokyo, Japan). Enzyme-linked immunosorbent assays were used to measure the blood concentrations of insulin (Mercodia, Sweden), active glucagon-like peptide-1 (GLP-1) (Millipore, Bedford, MA, USA), and GIP (Millipore). The homeostatic model assessment of insulin resistance (HOMA-IR) was calculated as the product of the fasting serum insulin concentration (in mU/L) and glucose concentration (in mg/dL), divided by 405.

#### Statistical analysis

Numerical data are expressed as means and standard deviations. A preliminary *F*-test of within-group variance homogeneity followed by Student’s *t*-test or a paired *t*-test was used when comparing values between the groups. A two-way repeated analysis of variance (ANOVA), followed by Bonferroni’s post hoc test, was used to compare changes over time and between the groups. Differences were considered significant when the error probability was <0.05. All statistical analyses were performed using GraphPad Prism 6 (GraphPad Software, La Jolla, CA, USA).

## Results

### Subject characteristics

The study participants were all male, with a mean age of 48.6 (standard deviation, SD: 5.8) years and a mean BMI of 25.4 (SD: 2.3) kg/m^2^ (Table 1). The concentrations of the blood parameters before each meal (in the fasting state) are presented in Table 2. There were no significant differences in these parameters between the test meal groups.

**Table 1:** Baseline demographic and anthropometric characteristics of the study subjects (mean values and standard deviation)

Index	Subjects (*n* = 19)	

	Mean	Standard deviation
Age (years)	48.6	5.8
Height (m)	1.71	0.05
Weight (kg)	74.9	8.5
Body mass index Ikg/m^2^)	25.4	2.3
% Body fat	22.1	3.6

**Table 2:** Concentrations of blood biomarkers obtained in the fasting state among the study subjects (mean values and standard deviation)

Index	Placebo (*n* = 19)		Rice bran triterpenoids (*n* = 19)	
	
	Mean	Standard deviation	Mean	Standard deviation
Glucose (mg/dL)	95.9	15.5	96.1	12.0
Insulin (mU/L)	5.95	3.06	5.76	2.80
Homeostasis model assessment of insulin resistance	1.39	0.74	1.37	0.69
Glucagon-like peptide 1 (pM)	3.48	11.43	1.40	3.19
Glucose-dependent insulinotropic polypeptide, (pg/mL)	64.8	34.5	55.9	26.0
Triglyceride (mg/dL)	145.7	78.2	154.2	75.5
Total cholesterol (mg/dL)	196.9	24.2	201.6	27.7
LDL-cholesterol (mg/dL)	118.2	17.5	126.1	17.6
HDL-cholesterol (mg/dL)	49.8	8.3	49.7	8.0
Aspartate aminotransferase (U/L)	23.5	9.4	23.5	10.3
Alanine aminotransferase (U/L)	29.8	19.1	31.1	21.1
Lactate dehydrogenase (U/L)	185.8	23.5	182.6	23.7

Note: There were no significant differences in the baseline variables between the test meal groups.

### Effects of RBTs on postprandial blood glucose levels

**Fig. 2:**
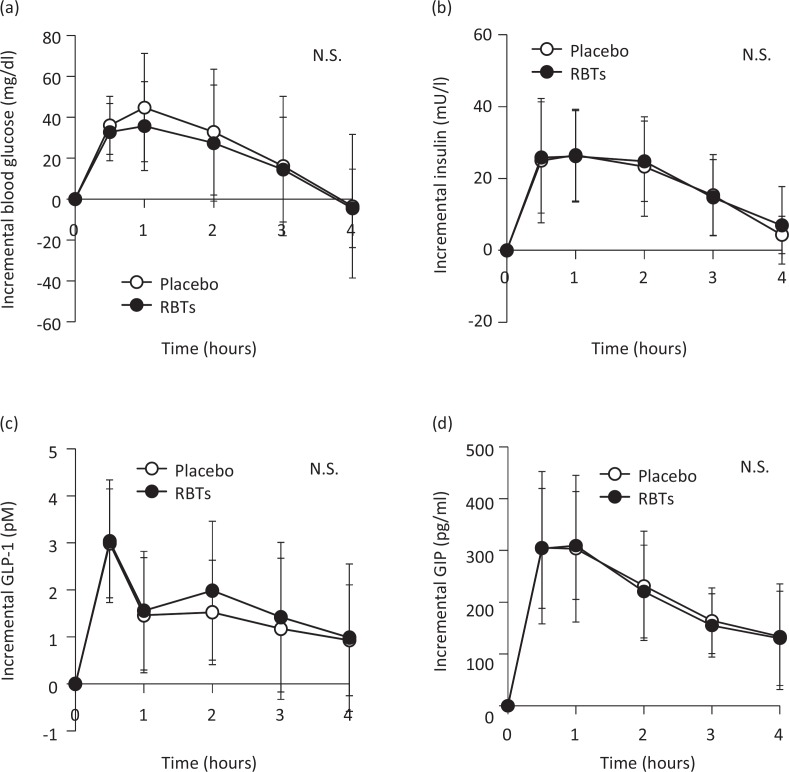
Postprandial changes in blood variables after the consumption of rice bran triterpenoids (RBTs). Blood glucose (a), insulin (b), active glucagon-like peptide-1 (GLP-1) (c), and total glucose-dependent insulinotropic polypeptide (GIP) (d) after the consumption of placebo (○, *n* = 19) and RBTs (●, *n* = 19). Data are expressed as means and standard deviations, which are represented by vertical bars. A two-way repeated analysis of variance was used to compare changes over time and between the groups.

The overall postprandial blood glucose responses tended to be lower (*P* = 0.087) after the RBT-supplemented meal relative to the placebo meal (Fig. 2a). The postprandial increases in blood glucose levels (area under the curve, AUC 0–1 h [*P* = 0.042], AUC 0–2 h [*P* = 0.014], or AUC 0–3 h [*P* = 0.012]) were significantly lower in subjects who consumed the RBT-supplemented meal, compared to the placebo meal (Table 3). No significant differences were observed in the postprandial blood insulin (Fig. 2b), GLP-1 (Fig. 2c), and GIP responses (Fig. 2d).

**Table 3:** Postprandial area under the curves for blood glucose concentration (mean values and standard deviations)

	Placebo (*n* = 19)		Rice bran triterpenoids (*n* = 19)		*P*
	
	Mean	Standard deviation	Mean	Standard deviation	
AUC_0–1 h_	29.2	11.9	25.3	10.4	0.042
AUC_0–2 h_	68.0	38.2	56.8	32.8	0.014
AUC_0–3 h_	92.4	66.3	77.7	57.8	0.012
AUC_0–4 h_	98.8	92.5	82.6	75.7	0.059

**Fig. 3:**
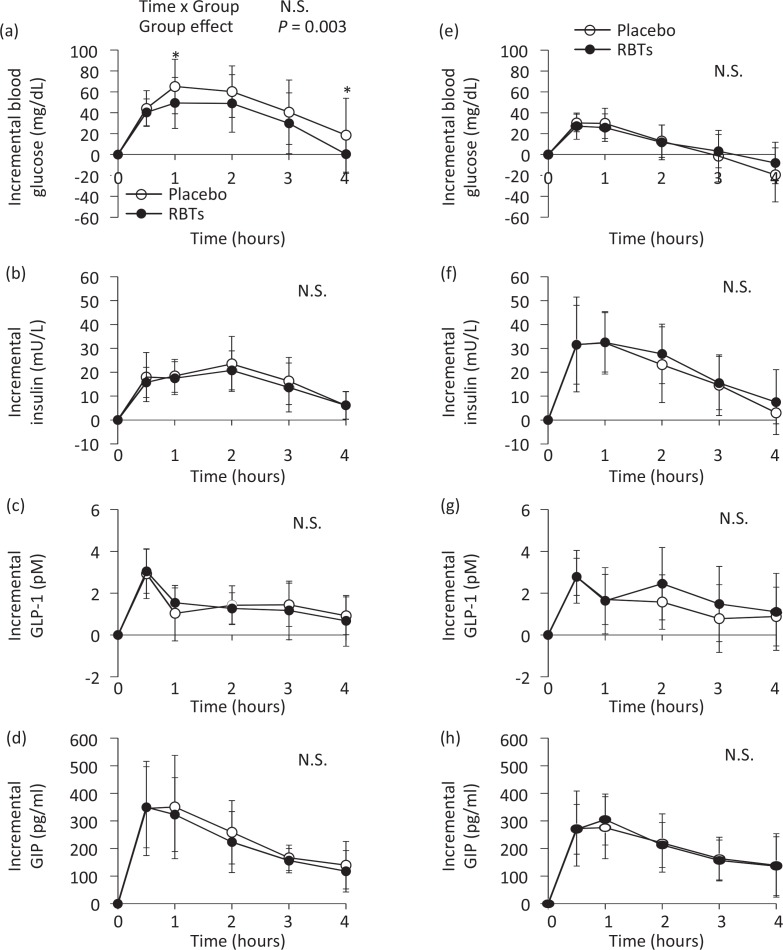
Postprandial changes in blood variables after rice bran triterpenoid (RBT) consumption in subjects with higher (a–d, *n =* 8) and lower (e–h, *n* = 11) postprandial incremental blood glucose levels at 2 h. Blood glucose (a, e), insulin (b, f), active glucagon-like peptide-1 (GLP-1) (c, g), and glucose-dependent insulinotropic polypeptide (GIP) (d, h) after the consumption of placebo (○) and RBTs (●) in subgroups comprising subjects with higher (>32.8 mg/dL; i.e. average of all subjects) or lower (<32.8 mg/dL) postprandial incremental blood glucose levels at 2 h after the placebo meal. Data are expressed as means and standard deviations, which are represented by vertical bars. A two-way repeated analysis of variance was used to compare changes over time and between the groups. Asterisks indicate the probability of random differences between groups: *P<0.05 indicates a significant difference between groups (Bonferroni test).

A subclass analysis of subjects (*n =* 8) with higher postprandial incremental blood glucose levels at 2 h after placebo meal ingestion (>32.8 mg/dL, the average of all subjects) showed significantly lower overall postprandial blood glucose levels (*P* = 0.003; Fig. 3a) and postprandial increases in blood glucose levels (AUC 0–1 h [*P* = 0.014], AUC 0–2 h [*P* = 0.002], AUC 0–3 h [*P*<0.001], or AUC 0–4 h [*P*<0.001]; Table 4) after consumption of the RBT-supplemented meal relative to the placebo meal. The fasting concentrations of blood variables did not differ between the placebo and RBT groups or between subjects with higher and lower postprandial glucose levels (Table 5).

**Table 4:** Postprandial area under the curves for blood glucose concentration in subjects with higher and lower postprandial glucose levels (mean values and standard deviations)

	Higher glucose level group (*n* = 8)					Lower glucose level group (*n* = 11)				
	
	Placebo		Rice bran triterpenoids		*P*	Placebo		Rice bran triterpenoids		*P*
			
	Mean	Standard deviation	Mean	Standard deviation		Mean	Standard deviation	Mean	Standard deviation	
AUC_0-1 h_	38.3	11.2	32.5	10.9	0.014	22.6	7.4	20.0	6.1	NS
AUC_0-2 h_	101.0	33.0	81.7	35.3	0.002	43.9	19.0	38.8	14.4	NS
AUC_0-3 h_	151.4	56.4	121.1	60.7	<0.001	49.6	29.9	46.2	28.8	NS
AUC_0-4 h_	180.9	72.1	136.2	78.8	<0.001	39.1	49.6	43.7	44.7	NS

NS, not significant.

**Table 5:** Concentrations of blood variables obtained in the fasting state among subjects with higher and lower postprandial glucose levels (mean values and standard deviation)

	Higher glucose level group (*n* = 8)				Lower glucose level group (*n* = 11)			
	
	Placebo		Rice bran triterpenoids		Placebo		Rice bran triterpenoids	
			
	Mean	Standard deviation	Mean	Standard deviation	Mean	Standard deviation	Mean	Standard deviation
Glucose (mg/dL)	96.0	9.9	97.5	12.2	95.7	19.0	95.1	12.3
Insulin (U/L)	5.23	1.97	4.96	1.24	6.47	3.67	6.34	3.49
Homeostasis model assessment of insulin resistance	1.21	0.43	1.18	0.31	1.52	0.90	1.51	0.86
Glucagon-like peptide 1 (pM)	0.67	0.79	0.52	0.59	5.51	14.97	2.04	4.13
Glucose-dependent insulinotropic polypeptide (pg/mL)	60.1	23.5	57.8	24.3	68.3	41.6	54.5	28.2
Triglyceride (mg/dL)	200.4	92.1	201.5	77.3	106.0	30.6	119.8	54.6
Total cholesterol (mg/dL)	214.9	16.6	217.5	26.2	183.8	20.3	190.1	23.4
LDL-cholesterol (mg/dL)	126.6	13.2	132.9	16.3	112.0	18.1	121.1	17.5
HDL-cholesterol (mg/dL)	51.7	9.8	51.7	10.3	48.4	7.1	48.2	6.0
Aspartate aminotransferase (U/L)	23.4	10.0	25.6	9.5	23.6	9.5	21.9	11.0
Alanine aminotransferase (U/L)	29.1	16.5	32.6	20.2	30.3	21.5	30.0	22.7
Lactate dehydrogenase (U/L)	191.4	22.6	188.0	21.0	181.7	24.3	178.6	25.8

Note: There were no significant differences in baseline variables between the test meal groups.

No significant differences were observed in the blood insulin (Fig. 3b), GLP-1 (Fig. 3c), and GIP responses (Fig. 3d). Among subjects (*n* = 11) with lower postprandial incremental blood glucose levels, all blood variables remained similar between the meal groups (Fig. 3e–h; Table 4).

## Discussion

This study indicates that the consumption of RBT-supplemented oil significantly decreases the postprandial increase in blood glucose levels in healthy male subjects, especially those with elevated postprandial hyperglycemia. Thirteen of the 19 subjects exhibited mitigated blood glucose increases after consuming RBT-supplemented oil, compared with the placebo. This finding supports our hypothesis that dietary RBT supplementation reduces postprandial hyperglycemia in healthy humans.

To our knowledge, this study is the first to report that the consumption of dietary oil supplemented with RBTs (16.5 mg, equivalent to approximately 3.7 g of rice bran) reduced the postprandial increase in the blood glucose level by >15%, compared with the control group, during a 240-min period after meal consumption; however, both groups maintained similar postprandial insulin responses. Our results, therefore, contradict the general perception that a decreased blood glucose response leads to a decreased postprandial insulin response. This contradiction might be explained by the finding that RBTs did not decrease the magnitude of the initial blood glucose increase, which may determine the initial release of insulin from pancreatic β cells. In fact, the increases in blood glucose levels during the first 30 min after meal ingestion were similar between the RBT and control groups. Accordingly, the consumption of RBTs is likely to reduce glucose levels beginning at 60 min after ingestion.

Our subclass analysis of subjects with elevated postprandial blood glucose responses also showed that the postprandial blood glucose response was significantly reduced by RBT consumption. Accordingly, dietary RBTs are likely to be effective even in subjects with impaired glucose tolerance or patients with diabetes.

Although RBT consumption significantly improved postprandial hyperglycemia, the postprandial GIP response was not affected. These results were inconsistent with our previous finding that RBTs reduced the high-fat diet-induced GIP response in mice (13). The reason for this difference remains unclear, but may be related to differences in the macronutrient compositions of the test meals. The carbohydrate:fat ratio of the test meal was 2:1 in our previous study (13), but was 20:1.1 (200 g of cooked rice with 11 g of olive oil) in the present study. Dietary fat is a more potent GIP secretagogue, compared to dietary carbohydrates. However, further studies are needed to clarify the discrepant findings that RBTs reduced the postprandial blood glucose response but not the GIP response after consuming a high-carbohydrate meal.

The major weakness of this study was the small sample size, particularly regarding subjects with higher postprandial blood glucose responses. Accordingly, it remains unclear whether consumption of the RBT-supplemented oil improved postprandial hyperglycemia. Further studies with larger sample sizes are warranted to clarify whether the consumption of RBT-supplemented oil could improve impaired glucose tolerance.

## Conclusion

In conclusion, the consumption of RBT-supplemented oil improved postprandial hyperglycemia in healthy human male subjects, especially those with higher postprandial increases in glucose levels. The consumption of oil-soluble RBTs might, therefore, benefit postprandial glycemic control. Further studies are needed to clarify the beneficial actions of RBTs in subjects with impaired glucose tolerance or patients with diabetes.
